# A Nutrient Formulation Affects Developmental Myelination in Term Infants: A Randomized Clinical Trial

**DOI:** 10.3389/fnut.2022.823893

**Published:** 2022-02-10

**Authors:** Nora Schneider, Muriel M. K. Bruchhage, Barry V. O'Neill, Mickaël Hartweg, Jérôme Tanguy, Pascal Steiner, Gisella Mutungi, Jonathan O'Regan, Séamus Mcsweeney, Viren D'Sa, Sean C. L. Deoni

**Affiliations:** ^1^Nestlé Institute of Health Sciences, Nestlé Research, Société des Produits Nestlé SA, Lausanne, Switzerland; ^2^Department of Pediatrics, Brown University, Providence, RI, United States; ^3^Rhode Island Hospital, Hasbro Children's Hospital, Providence, RI, United States; ^4^Department of Psychology, Stavanger University, Stavanger, Norway; ^5^Clinical Research Unit, Nestlé Research, Société des Produits Nestlé SA, Lausanne, Switzerland; ^6^Nestlé Nutrition, Société des Produits Nestlé SA, Vevey, Switzerland; ^7^Nestlé Development Centre Nutrition, Askeaton, Ireland

**Keywords:** infant nutrition, brain development, myelination, MRI, phospholipids

## Abstract

**Background and Objectives:**

Observational studies suggest differences between breast-fed and formula-fed infants in developmental myelination, a key brain process for learning. The study aims to investigate the efficacy of a blend of docosahexaenoic acid (DHA), arachidonic acid (ARA), iron, vitamin B12, folic acid, and sphingomyelin (SM) from a uniquely processed whey protein concentrate enriched in alpha-lactalbumin and phospholipids compared with a control formulation on myelination, cognitive, and behavioral development in the first 6 months of life.

**Methods:**

These are 6-month results from an ongoing two-center, randomized controlled trial with a 12-month intervention period (completed for all participants). In this study, full term, neurotypical infants of both sexes (*N* = 81) were randomized into investigational (*N* = 42) or control groups (*N* = 39). In addition, non-randomized breast-fed children (*N* = 108) serve as a natural reference group. Main outcomes are myelination (MRI), cognitive (Bayley Scales of Infant and Toddler Development, 3rd edition [Bayley-III]), social-emotional development (Ages and Stages Questionnaires: Social-Emotional, 2nd edition [ASQ:SE-2]), sleep (Brief Infant Sleep Questionnaire [BISQ]), and safety (growth and adverse events [AEs]).

**Results:**

The full analyses set comprises *N* = 66 infants. Significant differences in myelin structure, volume, and rate of myelination were observed in favor of the investigational myelin blend at 3 and 6 months of life. Effects were demonstrated for whole brain myelin and for cerebellar, parietal, occipital, and temporal regions, known to be functionally involved in sensory, motor, and language skills. No statistically significant differences were found for early behavior and cognition scores.

**Conclusions:**

This is the first study demonstrating the efficacy of a myelin nutrient blend in well-nourished, term infants on developmental myelination, which may be foundational for later cognitive and learning outcomes.

**Clinical Trial Registration:**

ClinicalTrials.gov, identifier: NCT03111927.

## Introduction

Myelination is a cornerstone of human neurodevelopment ensuring coordinated communication between brain cells and networks (1, 2). It has been positively associated with cognitive ability throughout postnatal development (~6.5–16 months) as well as in children and adults (3–5), making it an important research target for strategies that support brain, cognitive, and learning development.

Brain development is particularly rapid in the first years of life and requires nurturing care, such as nutrition (6, 7). Nutritional deficiencies, such as iron, vitamin B12, and folate, have been associated with hypomyelination, altered myelin composition, or decreased myelin synthesis (8–10). Observational studies suggest early life differences between formula-fed and breast-fed infants in fatty acid-related white matter (WM) composition (11) as well as in myelination and cognitive abilities, possibly linked to long-chain polyunsaturated fatty acids (LC-PUFA), sphingomyelin (SM), iron, and folic acid levels in infant nutrition (12, 13).

No intervention study has yet investigated the effects of nutritional supplementation on developmental (i.e., *de novo*) myelination. A pilot randomized clinical trial in low-birth-weight infants showed increased SM plasma and erythrocyte levels at 4, 6, and 8 weeks of life as well as improved scores in the behavior and attention measures at 18 months, following intervention with SM-fortified infant formula (20 vs. 13% of total milk phospholipids) in the first weeks of life (14). Myelination improvements were speculated by the authors as a potential mechanism.

A study in rodents using a diet containing different concentrations of docosahexaenoic acid (DHA) reported effects on myelin composition and function in pups in a concentration dependent manner (15). Similarly, we previously demonstrated that *in vitro* treatment with a blend of DHA, arachidonic acid (ARA), vitamin B12, folic acid, iron, and SM in a primary cell culture model increased the number of oligodendrocyte precursor cells, their differentiation, and maturation. These effects were dose-dependent with only higher doses of the blend showing significant effects. Furthermore, the data suggest a specific mechanistic impact of the individual nutrients and an interaction effect between the investigated nutrients with a positive net effect for the blend that was not observed for the individual compounds (16).

While available findings support the impact of nutrients on developmental myelination, their efficacy in term-born human infants has not been investigated. We conducted a two-center, double-blind, randomized controlled investigational trial in neurotypical term-born children to compare the effect of a nutrient blend containing DHA, ARA, iron, folic acid, vitamin B12, and SM (from a uniquely processed whey protein concentrate enriched in alpha-lactalbumin and phospholipids) in the first year of life to test the main hypotheses that higher levels of the nutrient blend increase myelin in the developing brain.

## Materials and Methods

### Trial Design

The study has a prospective, longitudinal, two-center (Memorial Hospital of Rhode Island and Pennington Biomedical Research Center, USA), double-blind, randomized, controlled, parallel group design with a 12-month intervention period and follow-up assessments up to 2 years of life. Five visits occur during the intervention period [V1 (6 ± 1 week of life), V2 (3 months ± 2 weeks), V3 (6 months ± 2 weeks), V4 (9 months ± 2 weeks), and V5 (12 months ± 2 weeks)]; two visits during the follow-up period [V6 (18 months ± 3 weeks), V7 (24 months ± 4 weeks)]. The follow-up visits are still ongoing while the 12-month intervention period has closed for all enrolled children. The reported findings refer to the staged statistical analysis of the 3- and 6-month time points.

### Trial Participants and Recruitment

Recruitment occurred between May 2017 and March 2020 *via* self-referral by one or more of the following: (1) local advertisements on social and general media, (2) information flyers and posters provided to community groups, and (3) information brochures given to women identified through or attending relevant hospital clinics.

Maternal screenings were performed during the third trimester of pregnancy up to and including post-delivery. Infants' enrollment window was between 2 and 5 weeks of life for formula-fed infants and breast-fed infants were enrolled earlier. Following written informed consent (screening visit), sociodemographic information, medical and family histories were collected, as well as a physical and neurological examination of the infant. Withdrawal from the study was possible at any point and with no further evaluations and any additional data collection. The research ethic boards at both clinical sites approved the protocol.

### Randomization and Blinding

Eligible non-breast-fed infants were randomized at V0 (baseline) into the investigational or control group with a 1:1 allocation using a permuted block randomization stratified by site and gender. Block size was not disclosed in the protocol to ensure the unpredictability of the randomization sequence. Investigational and control products were blinded by the manufacturer with non-speaking codes. Investigators, staff performing the assessments, and data analysts remain blind to the individual group allocation until the study database will be locked and the Statistical Analysis Plan (SAP) signed. To perform the statistical analysis of the data up to 6 months of age, a collaborator at the clinical site and an external contract research organization were unblinded. For the staged statistical analyses, only aggregated information was shared with the sponsor to ensure blinding until trial completion. Breast-fed children were not randomized.

### Trial Interventions

Intervention products were bovine milk-based infant formulas manufactured by Wyeth Nutrition, Askeaton, Ireland. The alpha-lactalbumin enriched whey protein concentrate used for the control product was almost devoid of phospholipids and SM, while the alpha-lactalbumin enriched whey protein concentrate used in the investigational product contained higher levels of SM and phospholipids due to the unique manufacturing process of ingredients. The investigational product contained higher levels of DHA, ARA, iron (fortified through ferrous sulfate heptahydrate), folic acid, and vitamin B12 (fortified through cyanocobalamine) (Table 1) than the control product. The higher levels were based on observational data and adjusted based on regulatory requirements for infant nutrition products. Nutrients were selected based on their reported presence in breast milk, use in infant formulas, and, in most cases, are mandated for inclusion as individual compounds in routine use infant formulas for healthy infants.

**Table 1:** Nutrient levels in intervention products^*^ and breast milk.

	**Investigational levels**	**Control levels**	**Breast milk levels (mean)** **V0**	**Breast milk levels (mean)** **V1**	**Breast milk levels (mean)** **V2**	**Breast milk levels (mean)** **Overall**
Energy (kcal per 200 ml serving)	132	132	/	/	/	/
Myelin blend						
Sphingomyelin (mg/L)	84	22	88	89	82	87
DHA (mg/L)	128	71	87	97	84	90
ARA (mg/L)	132	71	167	183	166	172
Iron (mg/L)	6.9	4.0	0.3	0.3	0.2	0.3
Folic acid (mcg/L)	175	85	24	27	23	25
B12 (mcg/L)	5.6	0.9	0.7	0.7	0.6	0.7
Phospholipids (mg/L)	410	186	282	286	268	277

*V0, 2–5 weeks of life; V1, 6 weeks ± 1 of life; V2, 3 months ± 2 weeks of life*.

* *Investigational product containing alpha-lactalbumin- and phospholipid-enriched whey protein concentrate; control product containing alpha-lactalbumin-enriched and phospholipid-depleted whey protein concentrate.*

Both blends were provided in an infant formula matrix and taken orally from enrollment to 12 months of life based on a response-feeding approach. Both intervention products included the same organoleptic and sensory characteristics to secure blinding. The investigational product contained the above described uniquely processed whey protein concentrate while the control product contained a standard whey protein concentrate.

The sponsor adheres to the WHO Code supporting exclusive breast-feeding for at least the first 6 months of life. The study team did not influence the parental feeding choice at any time. A lactation consultant was freely available to support and advise mothers regarding lactation.

### Trial Outcomes

The main outcomes for the staged statistical analyses were myelination measures derived from MRI at V2 (3 months) and V3 (6 months) following MRI acquisition and analyses applied in previous pediatric studies (2, 12, 17): myelin water fraction (MWF) for myelin volume (mcDESPOT) and T1 and T2 images for myelin structure (MP-RAGE).

Additional outcomes included overall cognitive development as assessed by the Bayley Scales of Infant and Toddler Development, 3rd edition (Bayley-III) (18) at V3 (6 months), social-emotional development as assessed by parent-report *via* Ages and Stages Questionnaires: Social-Emotional, 2nd edition (ASQ-SE:2) (19) at V2 (3 months) and V3 (6 months), brain volumetric outcomes as assessed by structural MRI (MP-RAGE) at V2 (3 months) and V3 (6 months), and infant sleep [Brief Infant Sleep Questionnaire (BISQ) (20)]. Descriptive measures included adverse events (AEs) and safety assessments.

### Data and Biological Sample Collection

Study data were collected by the trained research staff using an access-controlled, web-based database (electronic Case Report Form (eCRF), Medidata) within 7 days of each participant's visit. No personal information was stored in the study database but separately at each institution with access limited to study coordinators. The clinical data management systems developed for this study complied with the Good Clinical Practices (GCP) predicate rule requirements, laws, and regulations (Personal data protection) and allowed an audit of actions performed by users.

Maternal breast milk samples were collected at V0, V1, and V2 in the BF reference group. All breast milk analyses were carried out anonymously by accredited laboratories.

### Safety Assessment

Parent-reported AEs and infant growth measures for weight and length were collected. Medical history, AEs, and serious adverse events were coded according to the Medical Dictionary for Regulatory Activities (MedDRA) version 16.0.

### MR Imaging and Analyses

Neuroimaging was performed on a Siemens 3T Trio during natural non-sedated sleep [as shown in (21)]. mcDESPOT multicomponent relaxometry was used to derive MWF measures (22) and brain volumes for WM, gray matter (GM), cerebellum, and corpus callosum (CC). Whole brain, six *a priori* (cerebellum, frontal and parietal lobe, CC body, genu, and splenium), and two *post-hoc* (occipital and temporal lobe) WM regions of interest (ROI) were included.

Multicomponent relaxometry data were visually assessed for motion artifacts (such as blurring and ghosting) and MWF measures were estimated. The quantitative MWF images were non-linearly aligned to Montreal Neurological Institute (MNI) space using a multi-step, multi-scale approach. Brain regions with similar myelination patterns were identified by merging the 1,290 image volumes into a single 4D time-series (analogous to a functional imaging series) and using probabilistic independent component analyses (PICA) (23) to delineate the non-orthogonal but statistically independent spatial regions with shared temporal myelination trajectories in an unsupervised and data-driven manner. The number of extracted independent components (ICs) was derived from the estimate of Bayesian evidence. Previously calculated brain masks and initial WM, GM, and cerebral cerebrospinal fluid estimates were then aligned to individual anatomical data of each child and used as starting priors for the ANTs Atropos voxel-wise WM, GM, CC, and cerebellum segmentation method.

### Sample Size Justification

Based on previous data (2, 12, 17), the MWF served as the lead marker for sample size justification. With a power of 80%, a sample size of *n* = 64 completed infants per intervention group was estimated to cover a mean MWF difference between groups ≥ 0.005 (SD = 0.010). With an estimated dropout rate of 25%, a maximum of 86 infants per intervention group was planned to be enrolled. In total, 92 infants were planned to be enrolled in the non-randomized reference group of breast-fed infants, expecting a 30% drop-out rate. Sample size calculations were performed using SAS 9.3. Due to the operational constraints encountered by the clinical sites following the coronavirus disease 2019 (COVID-19) pandemic, the recruitment was stopped at 81 infants in the randomized arms and 108 infants in the breast-fed arm.

### Statistical Analysis

An SAP was developed prior to the staged statistical analysis of the 3- and 6-months data. All analyses were conducted according to the randomized assignment. Both intervention groups were compared using descriptive statistics, independent *t*-tests, as well as analysis of covariance (ANCOVA), corrected for stratification factors. Intercepts and slopes for longitudinal analyses were estimated using a mixed model for repeated measurements (MMRM) corrected for the chronological age of individuals. The *p*-values were used to indicate potentially interesting results and were not corrected for multiplicity. Endpoint distributions were evaluated, and planned analyses were adapted accordingly prior to the contract research organization unblinding.

*Post-hoc* analyses were performed to compare the non-randomized breast-fed reference group with the two randomized arms, providing descriptive statistics and inferential analyses conducted (superiority, two-sided) using ANCOVA corrected for the stratification factors and weighted by the estimated inverse propensity score (PS) of being breast-fed in the trial (1/PS for breast-fed infants and 1/(1-PS) for formula fed infants). The score was calculated with a logistic regression on breast-fed/not breast-fed explained by the baseline covariates maternal marital status, family income, maternal full scale IQ estimate (Wechsler Abbreviated Scale of Intelligence, 2nd edition, WASI-II), number of siblings, the highest level of education (mother, father, or any adult living with the child), the father living with the child, and maternal depression score (Edinburgh Postnatal Depression Scale, EPDS) at 6 weeks post-partum. When normality of the residues underlying the ANCOVA could not be assumed after data log-transformation, a non-parametrical Wilcoxon test was applied disregarding the propensity score.

## Results

### Participants

**Figure 1:**
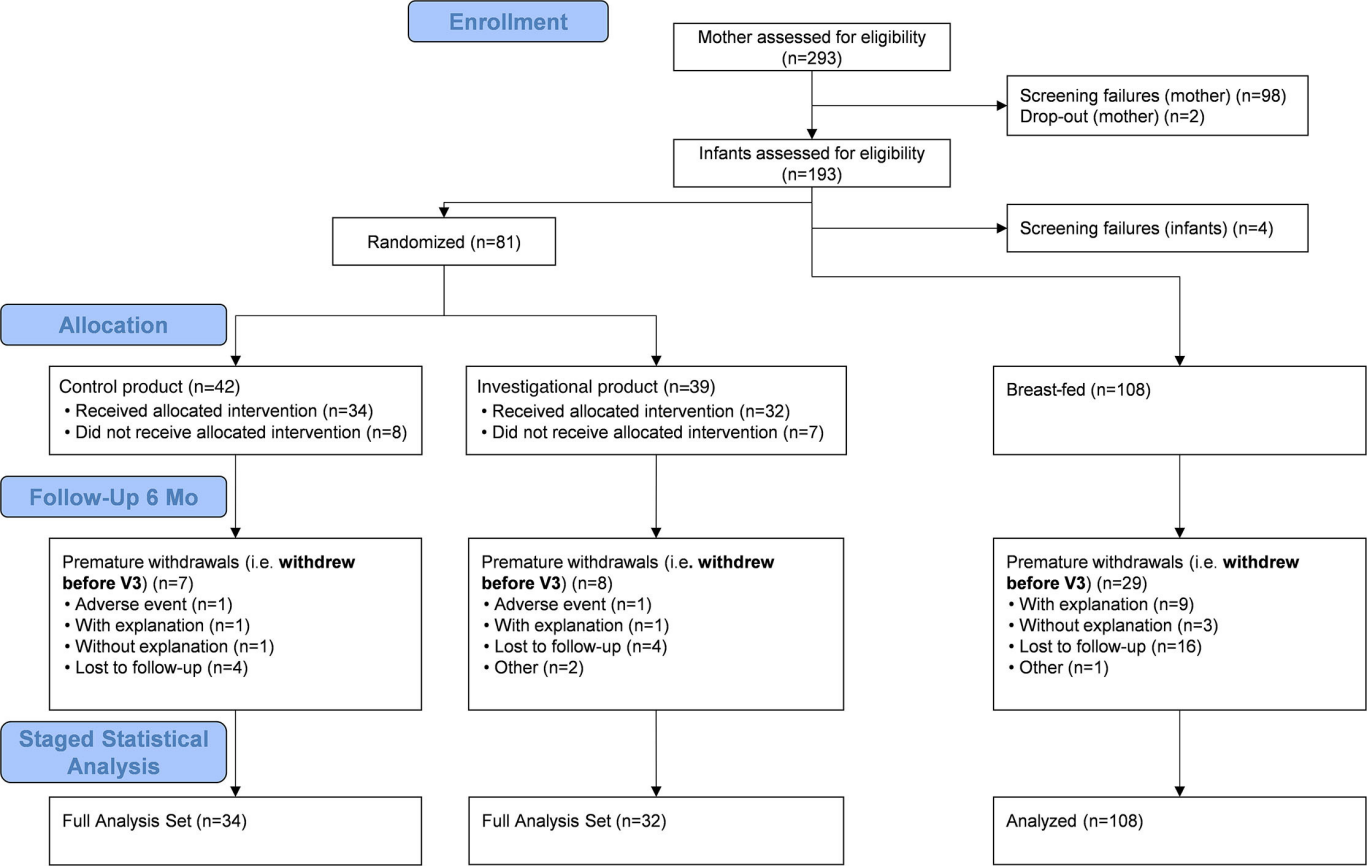
Flow diagram of participants included in the staged statistical analyses of the 3- and 6-months data. Children were randomized into one of two intervention groups, either receiving a blend of docosahexaenoic acid (DHA), arachidonic acid (ARA), iron, vitamin B12, folic acid, and sphingomyelin (SM) from a uniquely processed whey protein concentrate enriched in alpha-lactalbumin and phospholipids or a control formulation. Non-randomized breast-fed children served as a natural reference group.

A total of 189 infants were enrolled, of which 81 (42.9%) were randomized into the two intervention groups and 108 (57.1%) participated in the breast-feeding reference group. The mean infant age at enrollment was 29.9 (±7.45) days. Baseline characteristics were similar between intervention groups (Table 2). The full analysis set comprised 174 children (Figure 1). Reasons for premature withdrawal were evenly distributed between the randomized groups; missing data were considered as missing at random. Imaging data were available for 99 infants (56 girls) at 3 months and 71 infants (36 girls) at 6 months of age but reduced to 91 (52 girls) infants at 3 months, and 66 infants (34 girls) at 6 months of age for MWF and T1 data due to scanning complications at one of the sites (Table 3).

**Table 2:** Baseline characteristics of enrolled participants.

**Characteristics**	**Intervention group**		**Reference group**
	**Intervention (*N* = 39)**	**Control (*N* = 42)**	**Breast-fed (*N* = 108)**
**Children**			
Age at enrollment (days), mean (SD)	31.6 (7.84) *N*_available_ = 39	31.3 (6.06) *N*_available_ = 42	28.7 (7.64) N_available_ = 108
Gestational age (weeks), mean (SD)	38.8 (1.24) *N*_available_ = 39	39.1 (1.16) N_available_ = 42	39.3 (1.11) N_available_ = 108
Female sex, number (%)	18 (46.2%) *N*_available_ = 39	18 (42.9%) *N*_available_ = 42	61 (56.5%) *N*_available_ = 108
Weight at birth (kg), mean (SD)	3.27 (0.45) *N*_available_ = 39	3.27 (0.42) *N*_available_ = 42	3.43 (0.46) *N*_available_ = 108
Height at birth (cm), mean (SD)	49.89 (2.61) *N*_available_ = 37	50.19 (2.31) *N*_available_ = 39	50.37 (2.19) *N*_available_ = 104
Body fat (%), mean (SD)	17.87 (5.18) *N*_available_ = 39	17.32 (4.74) *N*_available_ = 40	17.46 (4.49) *N*_available_ = 107
**Number of siblings in same household**			
0 1–2 >3	14 (35.9%) 22 (56.4%) 3 (7.7%) *N*_available_ = 39	16 (38.1%) 22 (52.4%) 4 (9.5%) *N*_available_ = 42	39 (36.1%) 59 (54.6%) 10 (9.3%) *N*_available_ = 108
**Primary caregivers**			
Ethnicity mother	African-American 5 (12.8%) Asian 1 (2.6%) Caucasian 22 (56.4%) Mixed race 2 (5.1%) Other 9 (23.1%) *N*_available_ = 39	African-American 6 (14.3%) Caucasian 21 (50.0%) Mixed 5 (11.9%) Native American, Alaskan Native 2 (4.8%) Other 6 (14.3%) *N*_available_ = 40	African-American 8 (7.4%) Asian 3 (2.8%) Caucasian 76 (70.4%) Mixed race 8 (7.4%) Native American, Alaskan Native 1 (0.9%) Other 12 (11.1%) *N*_available_ = 108
Age (years), mean (SD)			
Mother Father	28.7 (5.58) *N*_available_ = 39 31.6 (6.95) *N*_available_ = 33	28.2 (4.95) *N*_available_ = 40 29.9 (7.31) *N*_available_ = 28	31.6 (4.84) *N*_available_ = 108 33.5 (5.52) *N*_available_ = 99
Maternal postnatal depression score at enrollment, mean (SD)	4.2 (3.74) N_available_ = 25	6.0 (4.47) N_available_ = 26	4.4 (4.09) N_available_ = 76
Maternal IQ, mean (SD)	96.4 (10.07) *N*_available_ = 39	97.0 (12.47) *N*_available_ = 41	104.1 (13.69) *N*_available_ = 108

**Table 3:** Participant demographics MRI data.

**Age distribution for MWF and T2**						
**Variable**	**Study group**	***N*** **available**	**Mean**	**SD**	**Minimum**	**Maximum**
Age (in months) at 3m	Control product	16	3.10	0.26	2.7	3.5
	Investigational product	13	2.98	0.32	2.6	3.5
	Breastfed	63	3.10	0.33	2.6	4.0
	All combined	92	3.08	0.32	2.6	4.0
Age (in months) at 6m	Control product	8	6.38	1.15	5.6	9.0
	Investigational product	10	6.24	0.90	5.7	8.0
	Breastfed	48	6.25	0.70	5.5	9.0
	All combined	66	6.26	0.78	5.5	9.0
**Age distribution for T1**						
**Variable**	**Study group**	***N*** **available**	**Mean**	**SD**	**Minimum**	**Maximum**
Age (in months)	Control product	20	3.06	0.26	2.7	3.5
	Investigational product	16	2.99	0.30	2.6	3.5
	Breastfed	63	3.10	0.33	2.6	4.0
	All combined	99	3.07	0.31	2.6	4.0
Age (in months) at 6 months	Control product	9	6.31	1.10	5.6	9.0
	Investigational product	14	6.07	0.80	5.6	8.0
	Breastfed	48	6.25	0.70	5.5	9.0
	All combined	71	6.22	0.77	5.5	9.0

*N available, Number of infants for whom data was available; SD, standard deviation; m, months; Investigational product, high level of selected nutrients; Control product, standard level of selected nutrients. MWF and T2 data are for the Resonance cohort only (including Breastfed group)*.

### Myelination

#### Myelin Volume and Structure

**Figure 2:**
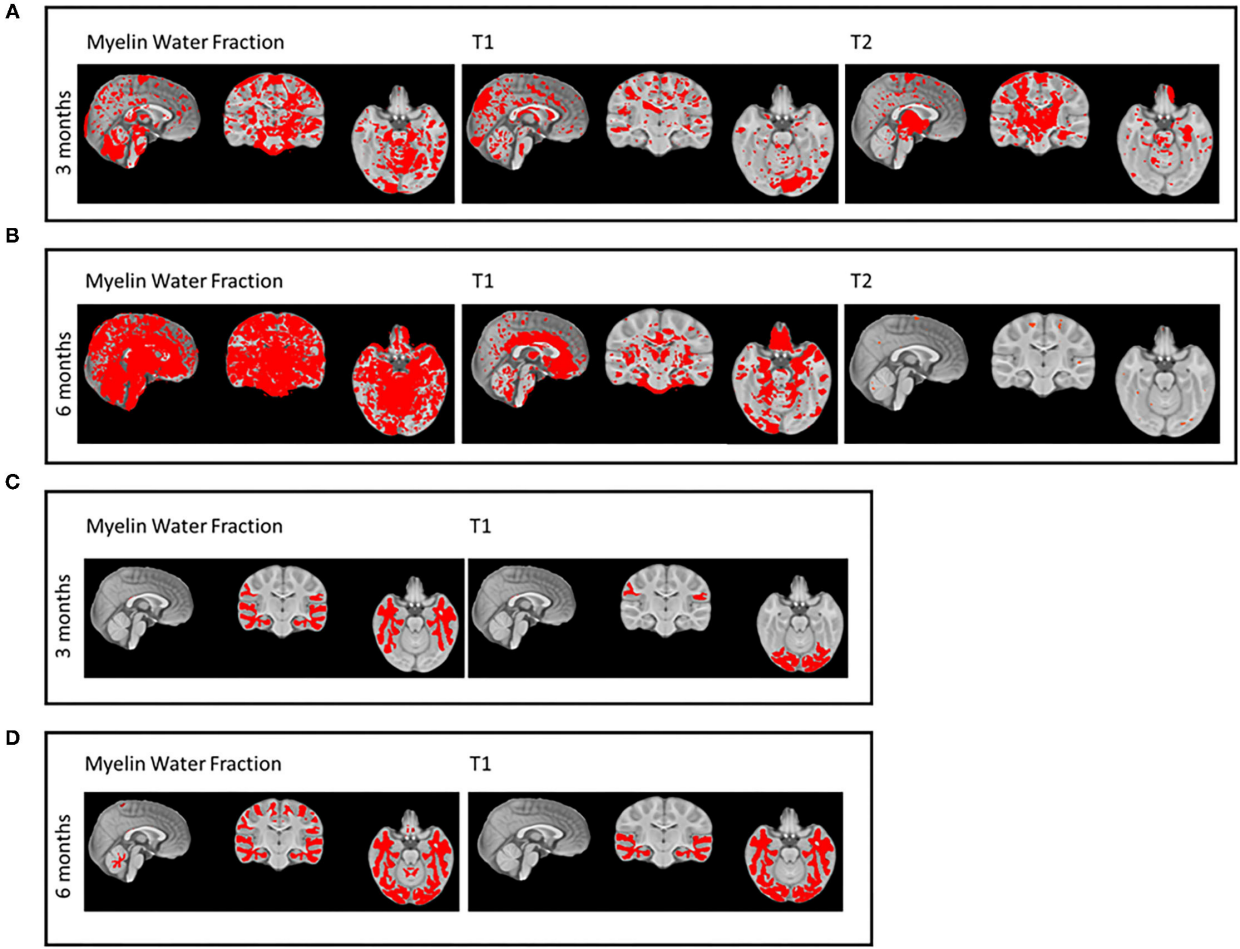
Intervention group differences in myelin volume (myelin water fraction [MWF]) and structure (T1 and T2) for independent component (IC) analyses **(A,B)** and for segmentation, such as cerebellum, frontal, and parietal lobe, corpus callosum (CC), occipital, and temporal lobe white matter (WM) regions **(C,D)**.

Of the 175 ICs identified (accounting for 80% of the total variance in the dataset), 24 parcellations showed significantly (*p* ≤.05, equal variances assumed) larger MWF volume, 19 significantly reduced T1, and 8 parcellations significantly reduced T2 values in the investigational over the control group at 3 months (Figure 2A). Seventy-nine parcellations showed significantly larger MWF volume, 15 significantly reduced T1, and one parcellation significantly reduced T2 values in the investigational over the control group at 6 months (Figure 2B). Regarding regions, the temporal lobe MWF was statistically larger, while occipital and parietal lobe T1 values were significantly reduced for the investigational group over the control group at 3 months of age. No significant differences were found for T2 scans (Figure 2C). At 6 months, the whole brain, cerebellum, parietal, occipital, and temporal lobe MWF were statistically larger and, occipital and temporal lobe T1 values were significantly smaller in the investigational over the control group. No significant differences were detected for T2 scans (Figure 2D). Descriptive statistics are shown in Supplementary Figures S1–S7.

*Post-hoc* analyses adjusted for the described propensity score indicated no statistically significant differences between the investigational and breast-fed groups nor between control and breastfeeding groups at 3 and 6 months for MWF of the whole brain and CC (splenium, body, and genu), for T2 of the whole brain, cerebellum, parietal and frontal WM, and CC (body and genu). Statistically significant lower T1 values of the whole brain and ROIs as well as higher MWF values of the cerebellum, parietal, and frontal WM were found in the investigational compared with the breast-fed group at 3 months but not at 6 months.

#### Myelin Growth Rate Differences

**Figure 3:**
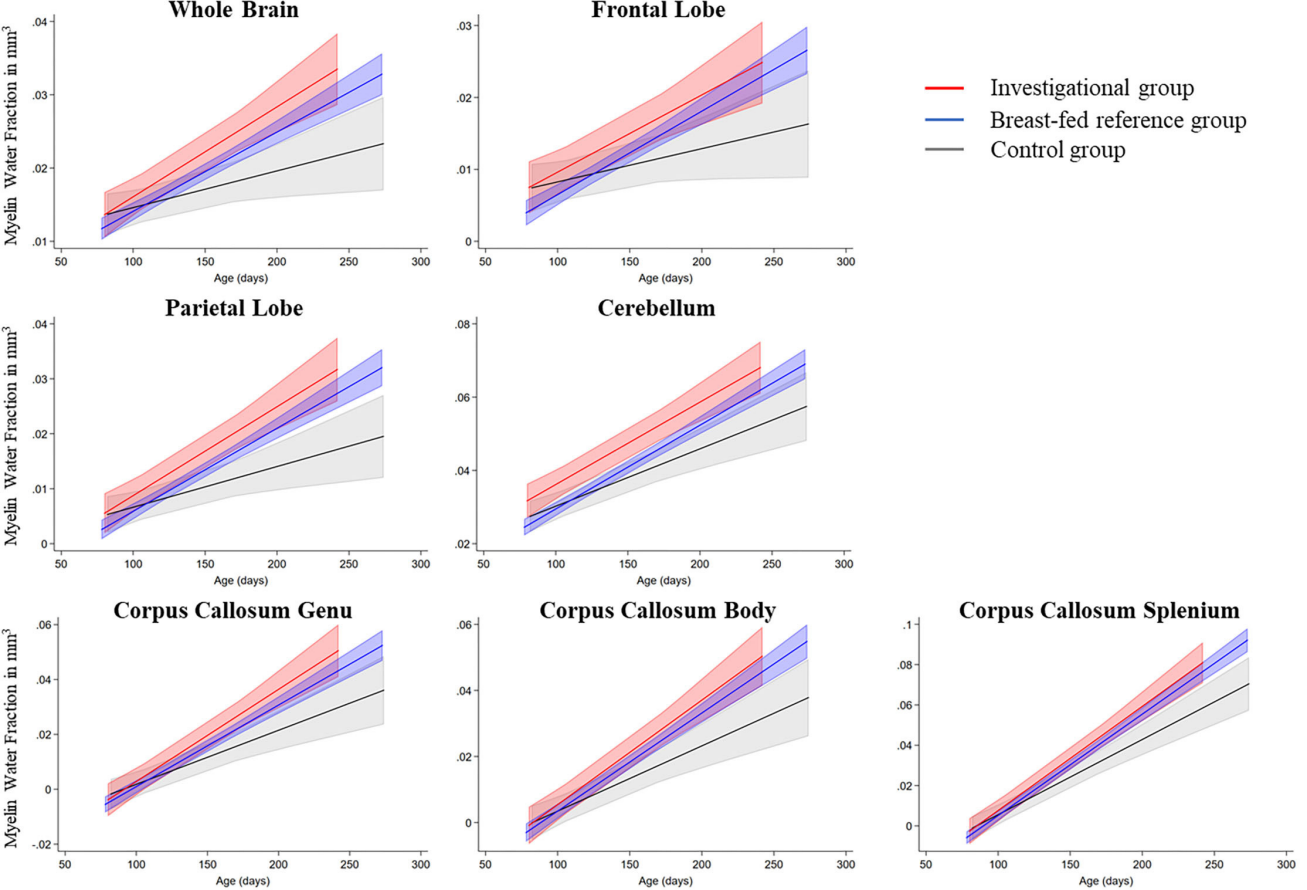
Myelin growth rate for the investigational, control, and breast-feeding reference group.

Longitudinal modeling of MWF slopes in the ROI yielded significantly steeper slopes in CC body (*p* = 0.025), CC genu (*p* = 0.016), CC splenium (*p* = 0.017), in temporal (*p* = 0.045), parietal (*p* = 0.012), and occipital lobes (*p* = 0.0511) as well as in the whole brain (*p* = 0.013) for the investigational group compared with control. No significant intercept differences were found between groups. MWF regression plots with confidence bands are displayed in Figure 3 and intercept and slope estimates in Table 4.

**Table 4:** Intercept and slope model estimates (myelin water fraction [MWF] in mm^3^).

**Region**	**Study group**	**Estimated intercept**	**Estimated slope**	*P* ^*^	**Estimated intercept difference (Inv. vs Control)**	*P* ^**^	**Estimated slope difference (Inv. vs Control)**	*P* ^**^
Cerebellum	Control	0.014596	0.000156	<0.0001	−0.000899	0.8786	0.000068	0.0984
Cerebellum	Investigational	0.013697	0.000225	<0.0001				
Cerebellum	Breast-fed	0.006631	0.000229	<0.0001				
Corpus callosum (body)	Control	−0.016324	0.000198	<0.0001	−0.009922	0.1850	0.000119	0.0250
Corpus callosum (body)	Investigational	−0.026246	0.000317	<0.0001				
Corpus callosum (body)	Breast-fed	−0.026274	0.000297	<0.0001				
Corpus callosum (genu)	Control	−0.017897	0.000197	<0.0001	−0.012825	0.1129	0.000139	0.0159
Corpus callosum (genu)	Investigational	−0.030722	0.000336	<0.0001				
Corpus callosum (genu)	Breast-fed	−0.028780	0.000298	<0.0001				
Corpus callosum (splenium)	Control	−0.031691	0.000373	<0.0001	−0.012265	0.1486	0.000144	0.0172
Corpus callosum (splenium)	Investigational	−0.043955	0.000516	<0.0001				
Corpus callosum (splenium)	Breast-fed	−0.045104	0.000503	<0.0001				
Whole brain (total)	Control	0.009594	0.000050	0.0162	−0.005785	0.1606	0.000073	0.0132
Whole brain (total)	Investigational	0.003809	0.000123	<0.0001				
Whole brain (total)	Breast-fed	0.003274	0.000108	<0.0001				
Frontal white matter	Control	0.003640	0.000046	0.0557	−0.004751	0.3191	0.000061	0.0704
Frontal white matter	Investigational	−0.001112	0.000107	<0.0001				
Frontal white matter	Breast-fed	−0.005102	0.000116	<0.0001				
Occipital white matter	Control	−0.005311	0.000150	<0.0001	−0.002739	0.6357	0.000080	0.0511
Occipital white matter	Investigational	−0.008050	0.000230	<0.0001				
Occipital white matter	Breast-fed	−0.013063	0.000225	<0.0001				
Parietal white matter	Control	−0.000766	0.000074	0.0027	−0.006642	0.1745	0.000088	0.0118
Parietal white matter	Investigational	−0.007408	0.000162	<0.0001				
Parietal white matter	Breast-fed	−0.009226	0.000151	<0.0001				
Temporal white matter	Control	−0.000625	0.000059	0.0062	−0.003571	0.4049	0.000061	0.0447
Temporal white matter	Investigational	−0.004196	0.000121	<0.0001				
Temporal white matter	Breast-fed	−0.005571	0.000103	<0.0001				

* *P, p-value against slope = 0*;

** *P, p-value for difference = 0*.

### Brain Volume, Behavior, and Safety

**Figure 4:**
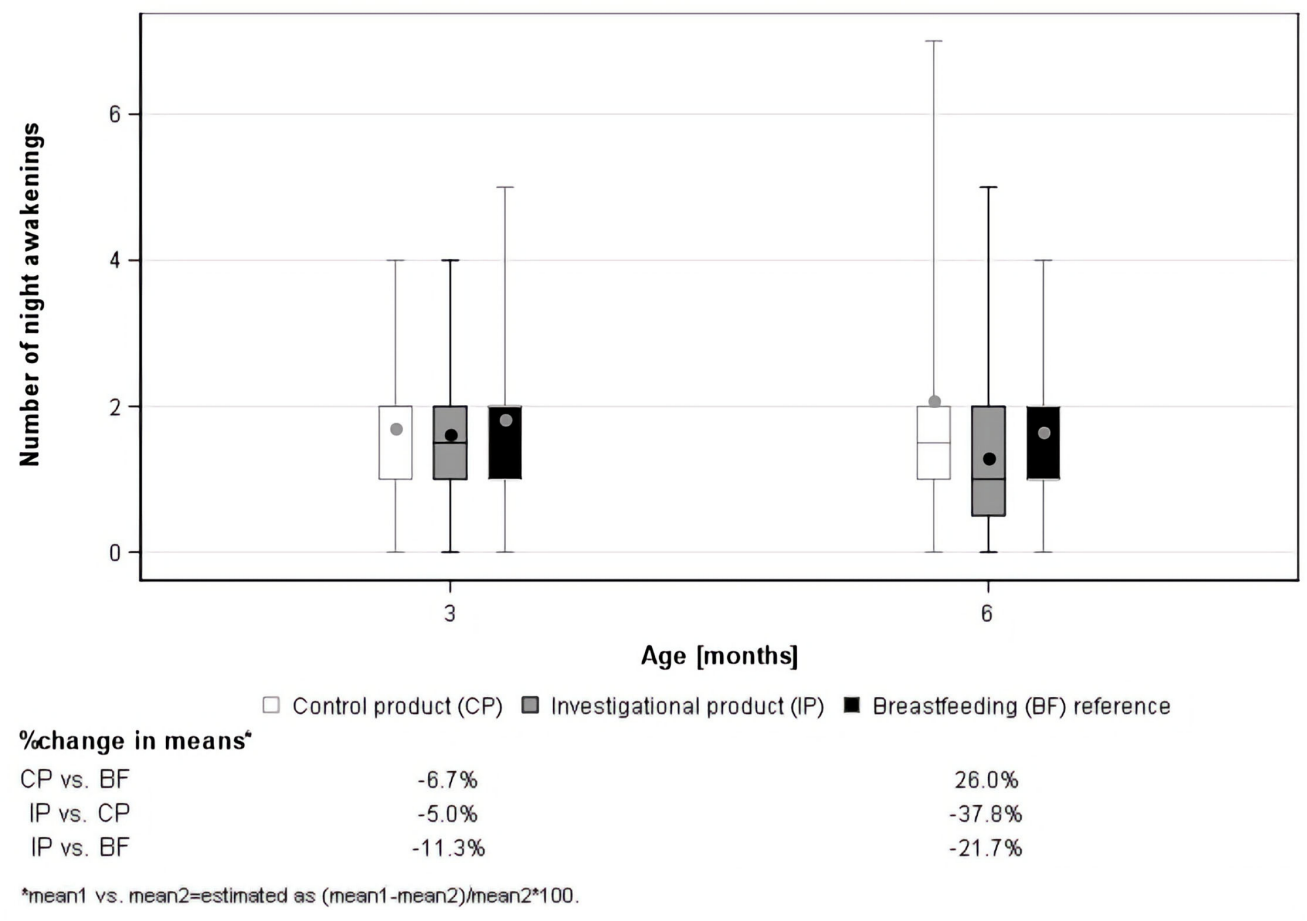
Boxplots of the number of night awakenings as assessed using the Brief Infant Sleep Questionnaire (BISQ).

No significant differences were found for total WM, GM, cerebellum, and CC volume, nor for cognitive, language, motor, social-emotional development, and sleep at 3 and 6 months. Results, however, showed a statistically non-significant reduction of night awakenings at 6 months in favor of the investigational product (least square means (LSMeans): −0.8 awakenings, 95% *CI* [−1.84; 0.24], *p* = 0.1262), with a least-square mean reduction of 38.6% compared with the control group (Figure 4).

Safety findings were largely similar across groups. Body weight and length values were between 10th and 90th percentiles for most infants at 3 (weight: 84, 89.3, and 83.7%; length: 80, 78.6, and 76.7% for the investigational, control, and breast-fed group, respectively) and 6 months (weight: 78.3, 74.1, and 74.7%; length: 82.6, 70.4, and 72.0% for the investigational, control, and breast-fed group, respectively). Regarding AE, 2/34 participants in the control group, 6/32 in the investigational group, and 3/108 in the breastfeeding group were reported to have had constipation. One AE in each group was considered related to the respective study product. No serious AE was reported.

## Discussion

This clinical trial is the first to demonstrate the impact of a myelin nutrient blend on developmental myelination in term-born infants as early as 3 and 6 months of life.

We demonstrated increases in myelin volume in favor of the investigational product for the whole brain as well as in parietal and temporal lobes at 3 months, and additionally in the cerebellum and occipital lobe at 6 months of age. These regions are known to be functionally involved in sensory, motor, cognitive, and language functions (24–26), such as inhibitory control over reflexive behaviors, target-directed head–eye coordination, reaching to grasp, and hand-to-hand transfer around 6 months of age (27).

The mean myelin volumes in our study for 3 (mean = 0.0148, SD = 0.0064) and 6 months (mean = 0.0233, SD = 0.0089) are in line with previously reported measures in larger observational cohort studies (28). The increased longitudinal MWF slopes in the whole brain, CC, parietal, and occipital regions possibly indicate age-appropriate and accelerated neurotypical development in the investigational myelin nutrient blend group. This is in line with neurotypical patterns of myelination, where primary sensory and motor areas myelinate before association areas [for review (29)]. Occipital and parietal lobe myelination has been placed by histological (30, 31) and neuroimaging studies (32) at 4 to 6 months of age. The *post-hoc* breast-feeding comparisons are for safety purposes and cannot be interpreted as efficacy data due to the limitations of comparing non-randomized arms. No statistically significant differences were identified for myelin volume (MWF) and myelin structure (T2) at 6 months. Some statistically significant differences were found for T1 at 3 months that disappear at 6 months, suggesting initial accelerated growth in the investigational group and a catch-up of the breast-fed group. Such potential early differences at 3 months could be related to maternal dietary intake and/or breast milk level differences for DHA, iron, folic acid, B12, and phospholipids, which were indeed lower in breast milk compared with the investigational product. Two of these, namely iron and folic acid, were in fact lower in breast milk than in the control product, which is in compliance with the United States guidelines for this type of product and target population. Without further data, biological relevance can, however, not be interpreted.

The absence of any measurable group differences in cognitive and behavioral outcomes this early in development appears in line with previous reports. The delayed cord clamping at birth was demonstrated to result in greater myelin content in some brain regions and greater ferritin levels at 4 (33) and 12 months (34) but no significant differences in the cognitive, motor, or language scores. Recent studies comparing enrichment vs. non-enrichment of certain lipid and protein compounds in infant nutrition (35), and comparing different levels of SM in infant formula (14) showed effects on the cognitive development at 1 and 1.5 years of age, respectively. These results suggest a potential temporal gap between intervention and functional changes. Longitudinal observational studies have consistently highlighted a latency between brain structural and behavioral benefits (3, 4, 26), leading to suggest later assessment time points to provide further insight on intervention effects.

The interim data indicate largely comparable findings across the three groups and a trend in slightly higher number of parent-reported constipation in the investigational group (18.8% compared with 5.9% in the control group and 2.8% in the breastfeeding group). A study in healthy term infants receiving standard control formula (*N* = 57) or formula enriched with a protein-rich milk fat globule membrane (MFGM) fraction (*N* = 72) or a lipid-predominant MFGM fraction (*N* = 70) up to 4 months of age reported constipation in 11.4% of the infants in the lipid-rich MFGM (compared with 9.7% in protein-rich MFGM group and 3.5% in standard control formula group) (36). To note, the adverse reports in the present study, should be interpreted with caution, and will be further monitored in the study.

Strengths of the study include the longitudinal RCT design, the breast milk reference group as well as the use of neuroimaging to investigate the impact of a nutritional blend on brain structural development and behavior.

Limitations include the impact of the COVID-19 pandemic on recruitment and retention, resulting in a smaller sample size than expected, which may have impacted possible treatment effects on the cognitive and behavioral variables. Environmental factors that may impact the brain and cognitive development, such as cognitive stimulation and parenting behavior, have not been investigated in the staged statistical analyses. For the full dataset at the study end, additional measures, such as day care attendance, parenting stress, and child activity levels will be available.

The investigational exploratory nature of the study requires cautious interpretation of the inferential statistics as type-I and type-II errors are uncontrolled. To mitigate this, the number of computed *p* was kept to a minimum. Lastly, the myelin imaging data were only analyzable from one of the two study sites due to acquisition issues with the myelin sequence. T1 and behavioral data were, however, unaffected and usable from both clinical sites.

Our findings add important insights into early brain architecture development. They highlight the opportunity to improve the developmental (i.e., *de novo*) myelination, a critical process in learning and development, *via* nutritional intervention in healthy infants.

## Data Availability Statement

The raw data supporting the conclusions of this article will be made available by the authors, without undue reservation.

## Ethics Statement

The studies involving human participants were reviewed and approved by Rhode Island Hospital and Pennington Biomedical Research Center Ethics Committees. Written informed consent to participate in this study was provided by the participants' legal guardian/next of kin.

## Author Contributions

NS, VD'S, and SD conceptualized and designed the study, drafted, and reviewed the manuscript. BO'N managed the project, wrote sections of the manuscript, reviewed, and revised it. MB and SD conducted the MRI data analyses, contributed to writing, reviewing, and revising the manuscript. JT and MH wrote the SAP, led the statistical activity of the trial and performed non-brain imaging data, contributed to writing, reviewing, and revising the manuscript. GM, JO'R, and SM led the nutritional and product activities, reviewed, and revised the manuscript. All authors contributed to the article and approved the submitted version.

## Funding

This study received funding from the Société des Produits Nestlé SA.

## Conflict of Interest

This study received funding from the Société des Produits Nestlé SA. The funder had the following involvement with the study: study design, data collection, and analysis, decision to publish, and preparation of the manuscript. NS, BO'N, MH, JT, PS, and GM were employed by Société des Produits Nestlé SA. JO'R and SM were employed by Nestlé Development Centre Nutrition, Ireland The remaining authors declare that the research was conducted in the absence of any commercial or financial relationships that could be construed as a potential conflict of interest.

## Publisher's Note

All claims expressed in this article are solely those of the authors and do not necessarily represent those of their affiliated organizations, or those of the publisher, the editors and the reviewers. Any product that may be evaluated in this article, or claim that may be made by its manufacturer, is not guaranteed or endorsed by the publisher.

## Supplementary Material

Supplementary Figure S1 Descriptive statistics for myelin volume and structure in the corpus callosum (CC) splenium.

Supplementary Figure S2 Descriptive statistics for myelin volume and structure in the whole brain.

Supplementary Figure S3 Descriptive statistics for myelin volume and structure in the cerebellum.

Supplementary Figure S4 Descriptive statistics for myelin volume and structure in parietal white matter (WM).

Supplementary Figure S5 Descriptive statistics for myelin volume and structure in frontal WM.

Supplementary Figure S6 Descriptive statistics for myelin volume and structure in the corpus callosum (CC) genu.

Supplementary Figure S7 Descriptive statistics for myelin volume and structure in the corpus callosum (CC) body.

## References

[1] Barkovich AJ. Magnetic resonance techniques in the assessment of myelin and myelination. J Inherit Metab Dis. (2005) 28:311–43. 10.1007/s10545-005-5952-z 15868466

[2] Dean DC III O'Muircheartaigh J Dirks H Waskiewicz N Lehman K Walker L. Modeling healthy male white matter and myelin development: three through 60 months of age. Neuroimage. (2014) 84:742–52. 10.1016/j.neuroimage.2013.09.058 24095814 PMC3895775

[3] Dai X Hadjipantelis P Wang JL Deoni SCL Müller HG. Longitudinal associations between white matter maturation and cognitive development across early childhood. Hum Brain Mapp. (2019) 40:4130–45. 10.1002/hbm.24690 31187920 PMC6771612

[4] Deoni SC O'Muircheartaigh J Elison JT Walker L Doernberg E Waskiewicz N. White matter maturation profiles through early childhood predict general cognitive ability. Brain Struct Funct. (2014) 221:1189–203. 10.1007/s00429-014-0947-x 25432771 PMC4771819

[5] Scholz J Klein MC Behrens TE Johansen-Berg H. Training induces changes in white-matter architecture. Nat Neurosci. (2009) 12:1370. 10.1038/nn.2412 19820707 PMC2770457

[6] Daelmans B Darmstadt GL Lombardi J Black MM Britto PR Lye S. Early childhood development: the foundation of sustainable development. Lancet. (2017) 389:9–11. 10.1016/S0140-6736(16)31659-2 27717607

[7] World Health Organization Unicef World Bank Group. Nurturing Care For Early Childhood Development: A Framework For Helping Children Survive and Thrive to Transform Health And Human Potential. Geneva: World Health Organization (2018).

[8] Prado EL Dewey KG. Nutrition and brain development in early life. Nutr Rev. (2014) 72:267–84. 10.1111/nure.12102 24684384

[9] Black RE Allen LH Bhutta ZA Caulfield LE De Onis M Ezzati M. Maternal and child undernutrition: global and regional exposures and health consequences. Lancet. (2008) 371:243–60. 10.1016/S0140-6736(07)61690-0 18207566

[10] Lozoff B Georgieff MK editors. Iron deficiency and brain development. Sem Pedia Neurol. (2006) 3:158–165: Elsevier. 10.1016/j.spen.2006.08.004 17101454

[11] Jamieson E Farquharson J Logan R Howatson A Patrick W Weaver L. Infant cerebellar gray and white matter fatty acids in relation to age and diet. Lipids. (1999) 34:1065–71. 10.1007/s11745-999-0458-5 10580334

[12] Deoni S Dean D III Joelson S O'Regan J Schneider N. Early nutrition influences developmental myelination and cognition in infants and young children. Neuroimage. (2017) 178:649–59. 10.1016/j.neuroimage.2017.12.056 29277402 PMC6540800

[13] Schneider N Hauser J Oliveira M Cazaubon E Mottaz SC O'Neill BV. Sphingomyelin in brain and cognitive development: preliminary data. eNeuro. (2019) 6:1–13. 10.1523/ENEURO.0421-18.2019 31324675 PMC6709232

[14] Tanaka K Hosozawa M Kudo N Yoshikawa N Hisata K Shoji H. The pilot study: sphingomyelin-fortified milk has a positive association with the neurobehavioural development of very low birth weight infants during infancy, randomized control trial. Brain Dev. (2013) 35:45–52. 10.1016/j.braindev.2012.03.004 22633446

[15] Haubner L Sullivan J Ashmeade T Saste M Wiener D Carver J. The effects of maternal dietary docosahexaenoic acid intake on rat pup myelin and the auditory startle response. Dev Neurosci. (2007) 29:460–7. 10.1159/000107047 17684314

[16] Hauser J Sultan S Rytz A Steiner P Schneider N A blend containing docosahexaenoic acid arachidonic acid vitamin B12 vitamin B9 iron and sphingomyelin promotes myelination in an in vitro model. Nutr Neurosci. (2020) 23:931–45. 10.1080/1028415X.2019.1580918 30806182

[17] Deoni SC Dean III DC Piryatinsky I O'Muircheartaigh J Waskiewicz N Lehman K. Breastfeeding and early white matter development: a cross-sectional study. Neuroimage. (2013) 82:77–86. 10.1016/j.neuroimage.2013.05.090 23721722 PMC3777218

[18] NB NB Bayley Scales of Infant and Toddler Development® (Bayley-III®). 3rd ed. San Antonio, TX: Pearson (2005). 10.1037/t14978-000

[19] Squires J Bricker D Twombly E. Ages & Stages Questionnaires: Social-Emotional: Paul H. Baltimore: Brookes Publishing Company Baltimore. (2002). 10.1037/t11524-000

[20] Sadeh AA. brief screening questionnaire for infant sleep problems: validation and findings for an Internet sample. Pediatrics. (2004) 113:e570–e7. 10.1542/peds.113.6.e570 15173539

[21] Bruchhage MMK Ngo GC Schneider N D'Sa V Deoni SCL. Functional connectivity correlates of infant and early childhood cognitive development. Brain Struct. Funct. (2020) 225:669–81. 10.1007/s00429-020-02027-4 32060640 PMC7046571

[22] Deoni SC Matthews L Kolind SH. One component? Two components? Three? The effect of including a nonexchanging “free” water component in multicomponent driven equilibrium single pulse observation of T1 and T2. Mag Res Med. (2013) 70:147–54. 10.1002/mrm.24429 22915316 PMC3711852

[23] Beckmann CF Smith SM. Probabilistic independent component analysis for functional magnetic resonance imaging. IEEE Trans Med Imaging. (2004) 23:137–52. 10.1109/TMI.2003.822821 14964560

[24] Fogassi L Luppino G. Motor functions of the parietal lobe. Curr Opin Neurobiol. (2005) 15:626–31. 10.1016/j.conb.2005.10.015 16271458

[25] Chang TT Metcalfe AW Padmanabhan A Chen T Menon V. Heterogeneous and non-linear development of human posterior parietal cortex function. Neuroimage. (2016) 126:184–95. 10.1016/j.neuroimage.2015.11.053 26655682

[26] O'Muircheartaigh J Dean DC III Ginestet CE Walker L Waskiewicz N Lehman K. White matter development and early cognition in babies and toddlers. Hum Brain Mapp. (2014) 35:4475–87. 10.1002/hbm.22488 24578096 PMC4336562

[27] Tau GZ Peterson BS. Normal development of brain circuits. Neuropsychopharmacology. (2010) 35:147–68. 10.1038/npp.2009.115 19794405 PMC3055433

[28] Dean DC O'Muircheartaigh J Dirks H Waskiewicz N Walker L Doernberg E. Characterizing longitudinal white matter development during early childhood. Brain Stru Fun. (2015) 220:1921–33. 10.1007/s00429-014-0763-3 24710623 PMC4481335

[29] Silbereis JC Pochareddy S Zhu Y Li M Sestan N. The cellular and molecular landscapes of the developing human central nervous system. Neuron. (2016) 89:248–68. 10.1016/j.neuron.2015.12.008 26796689 PMC4959909

[30] Kinney HC Brody BA Kloman AS Gilles FH. Sequence of central nervous system myelination in human infancy. II Patterns of myelination in autopsied infants. J Neuropathology Expe Neuro. (1988) 47:217–34. 10.1097/00005072-198805000-00003 3367155

[31] Yakovlev P Lecours A. The myelogenetic cycles of regional maturation of the brain. Minkowski A, editor Oxford: Blackwell (1967).

[32] Deoni SC Mercure E Blasi A Gasston D Thomson A Johnson M. Mapping infant brain myelination with magnetic resonance imaging. J Neurosci. (2011) 31:784–91. 10.1523/JNEUROSCI.2106-10.2011 21228187 PMC6623428

[33] Mercer JS Erickson-Owens DA Deoni SCL Dean DC III Collins J Parker AB. Effects of delayed cord clamping on 4-month ferritin levels, brain myelin content, and neurodevelopment: a randomized controlled trial. J Pedi. (2018) 203:266–72.e2. 10.1016/j.jpeds.2018.06.006 30473033 PMC6259583

[34] Mercer JS Erickson-Owens DA Deoni SCL Dean Iii DC Tucker R Parker AB. The effects of delayed cord clamping on 12-month brain myelin content and neurodevelopment: a randomized controlled trial. Am J Perinatol. (2020) 39:37–44. 10.1055/s-0040-1714258 32702760 PMC9800052

[35] Li F Wu SS Berseth CL Harris CL Richards JD Wampler JL. Improved neurodevelopmental outcomes associated with bovine milk fat globule membrane and lactoferrin in infant formula: a randomized, controlled trial. J Pedi. (2019) 215:24–31.e8. 10.1016/j.jpeds.2019.08.030 31668885

[36] Billeaud C Puccio G Saliba E Guillois B Vaysse C Pecquet S. Safety and tolerance evaluation of milk fat globule membrane-enriched infant formulas: a randomized controlled multicenter non-inferiority trial in healthy term infants. Clin Med Insig Pedi. (2014) 8:51–60. 10.4137/CMPed.S16962 25452707 PMC4219856

