# Supplementary figures and images for: A Nutrient Formulation Affects Developmental Myelination in Term Infants: A Randomized Clinical Trial
Source: Front Nutr. 2022 Feb 10;9:823893. doi: 10.3389/fnut.2022.823893 (PMC8886575; doi:10.3389/fnut.2022.823893)

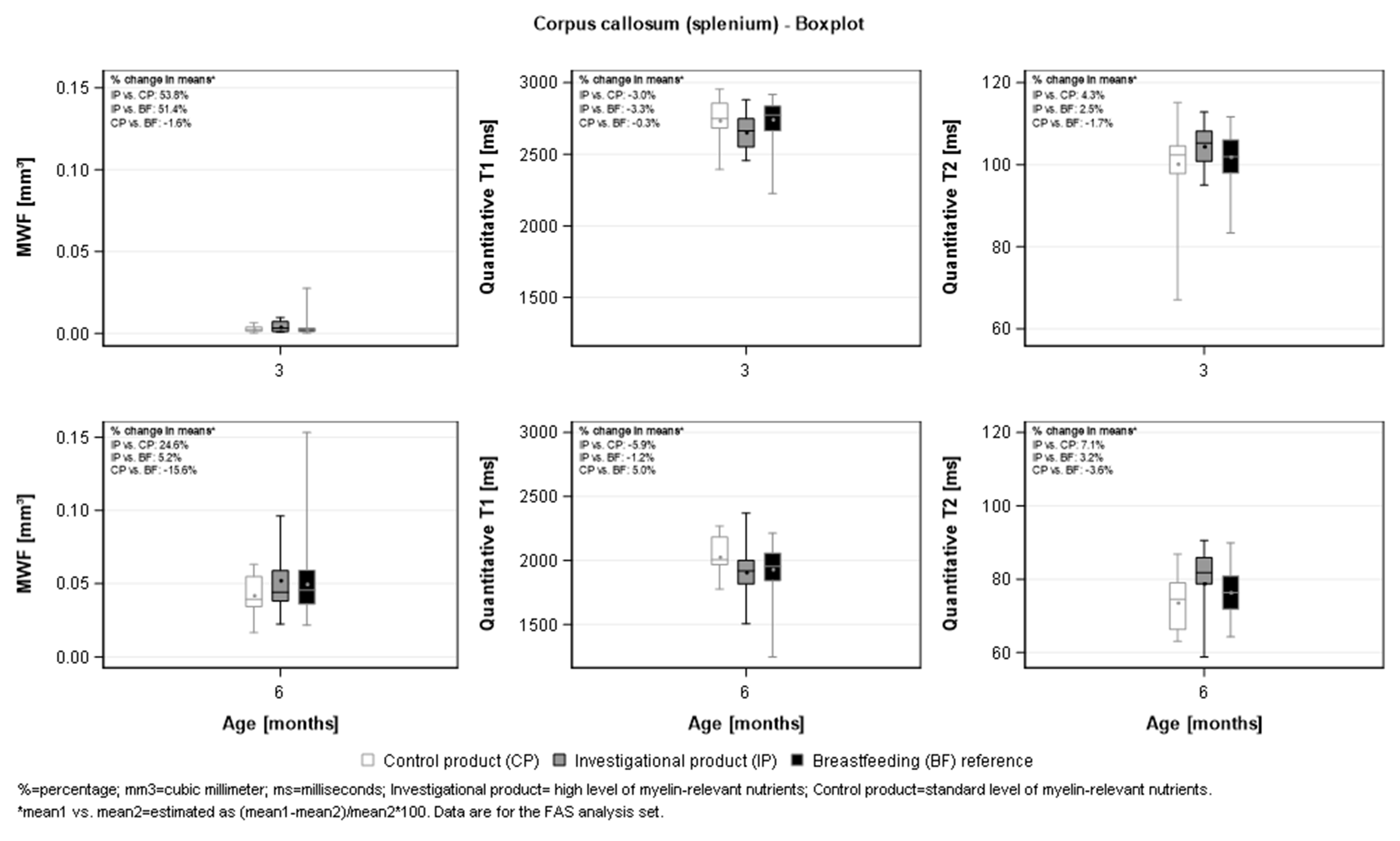

Supplement: Supplementary Figure S1 — Descriptive statistics for myelin volume and structure in the corpus callosum (CC) splenium. [file Image_1.JPEG]

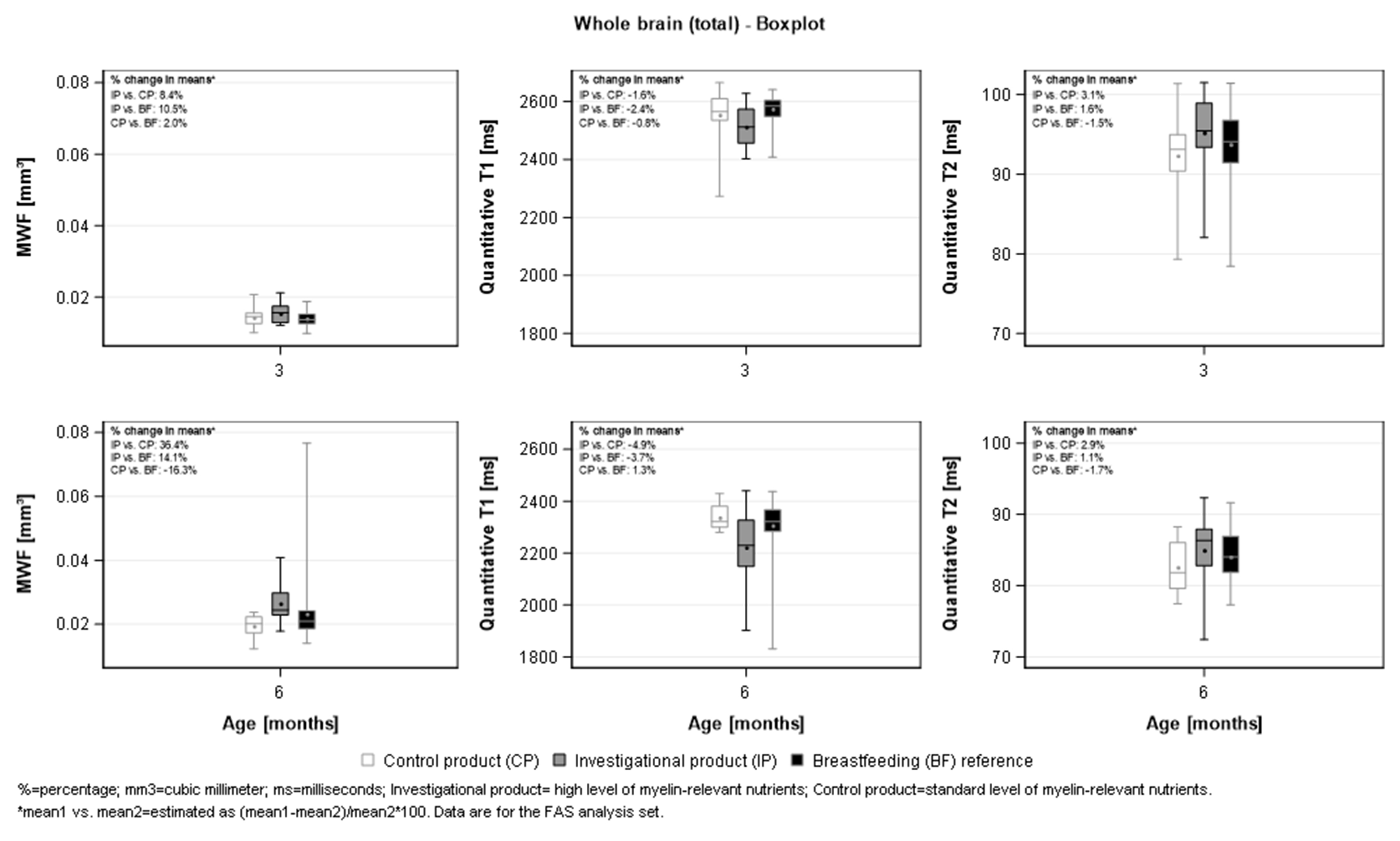

Supplement: Supplementary Figure S2 — Descriptive statistics for myelin volume and structure in the whole brain. [file Image_2.JPEG]

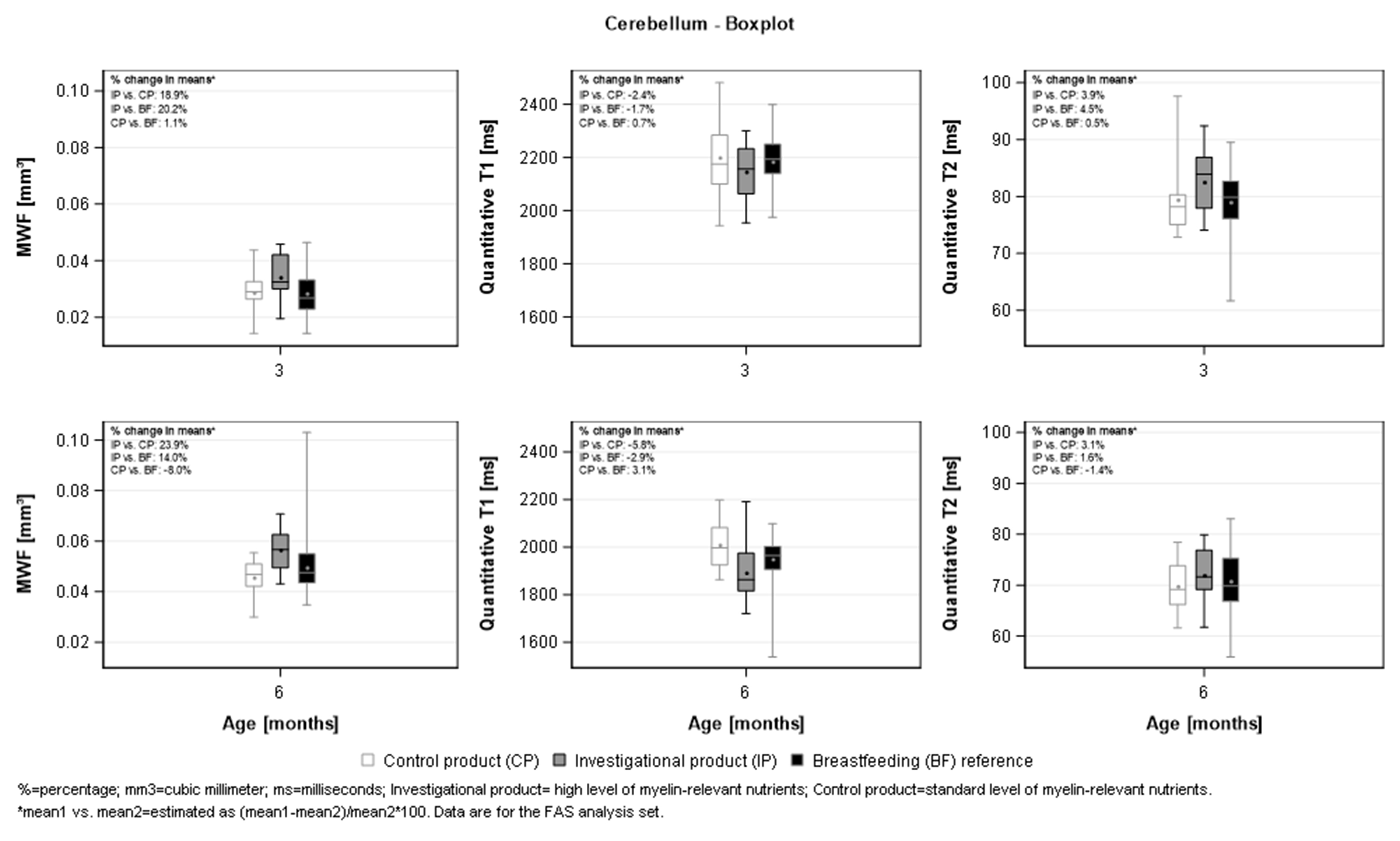

Supplement: Supplementary Figure S3 — Descriptive statistics for myelin volume and structure in the cerebellum. [file Image_3.JPEG]

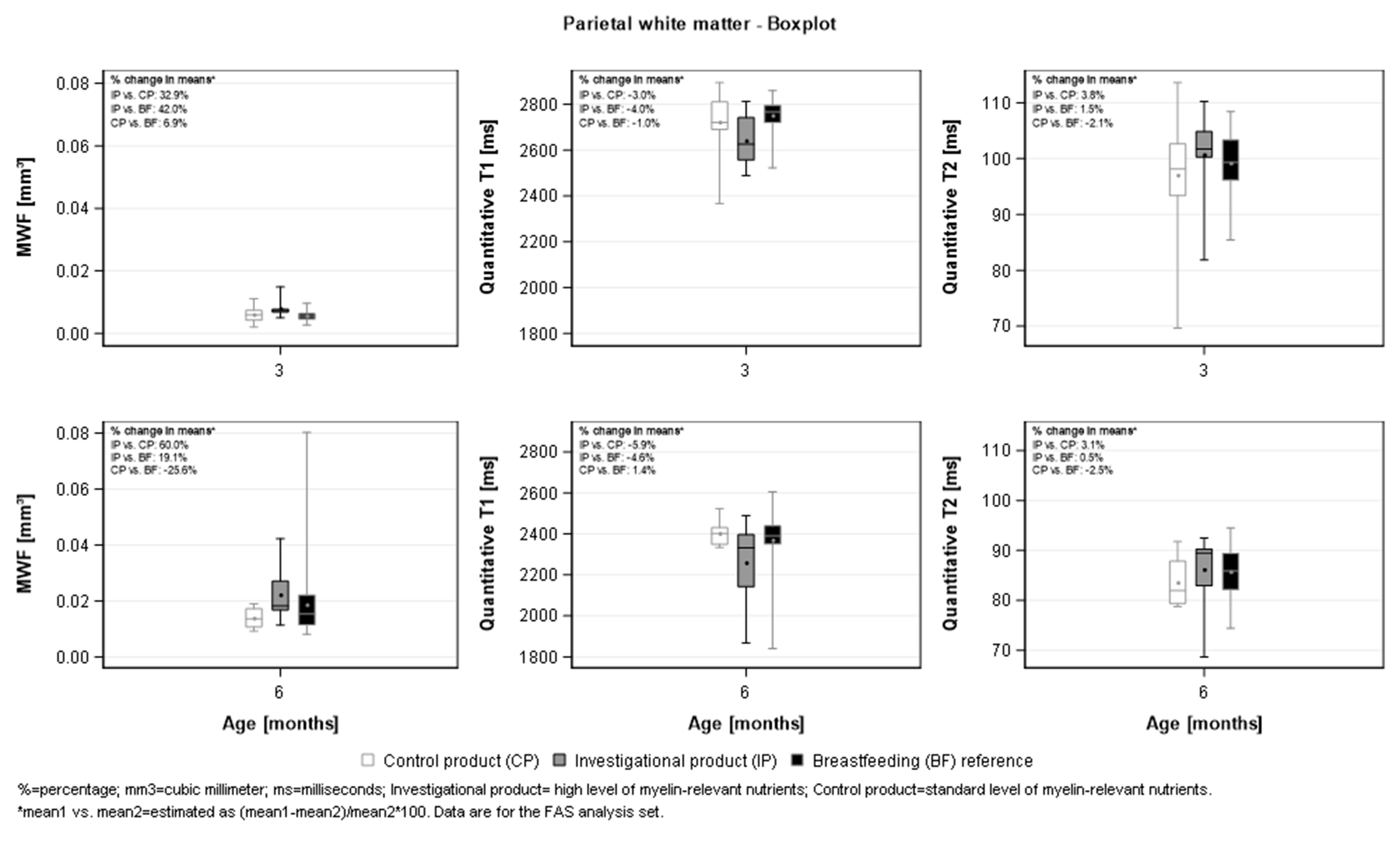

Supplement: Supplementary Figure S4 — Descriptive statistics for myelin volume and structure in parietal white matter (WM). [file Image_4.JPEG]

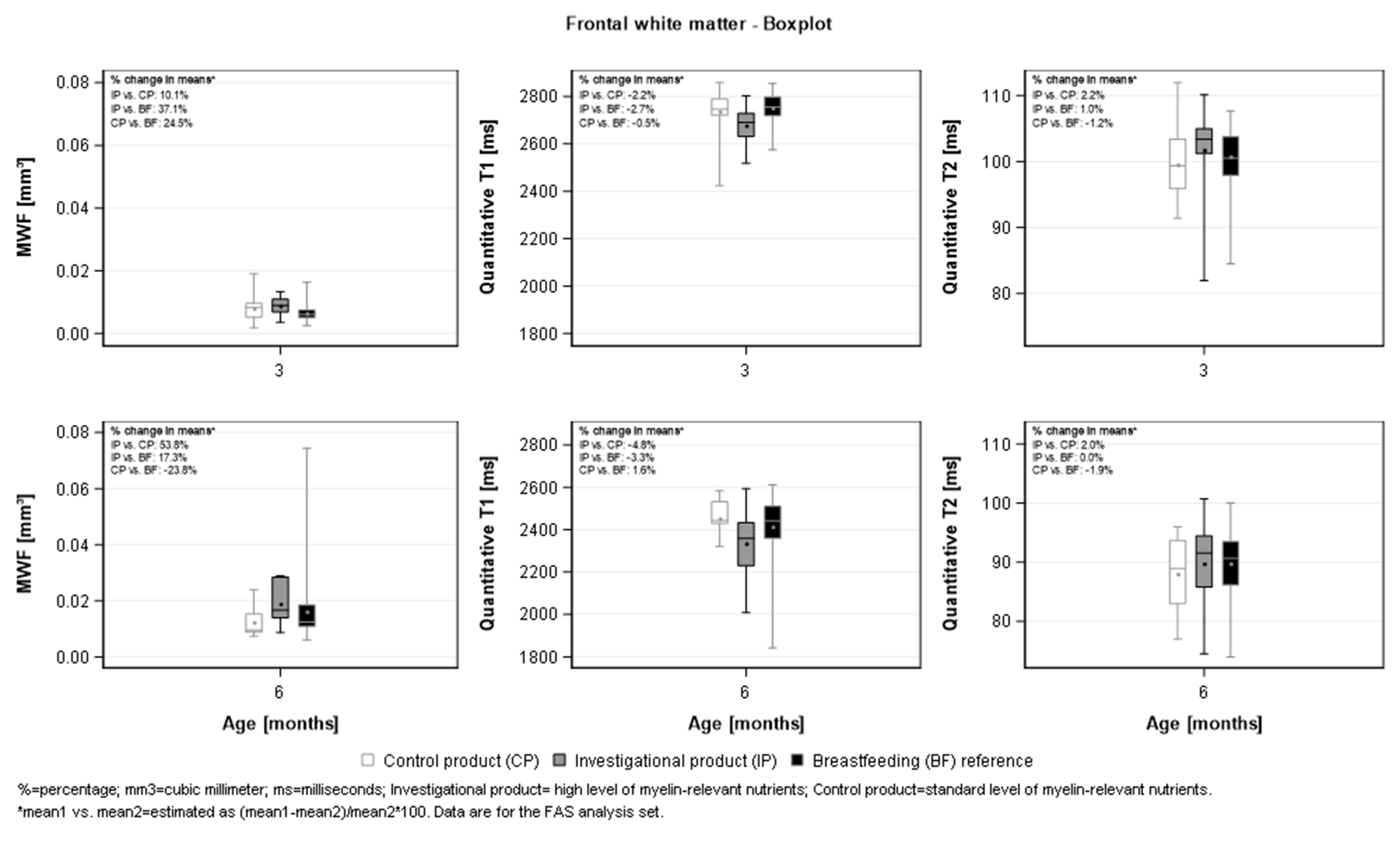

Supplement: Supplementary Figure S5 — Descriptive statistics for myelin volume and structure in frontal WM. [file Image_5.JPEG]

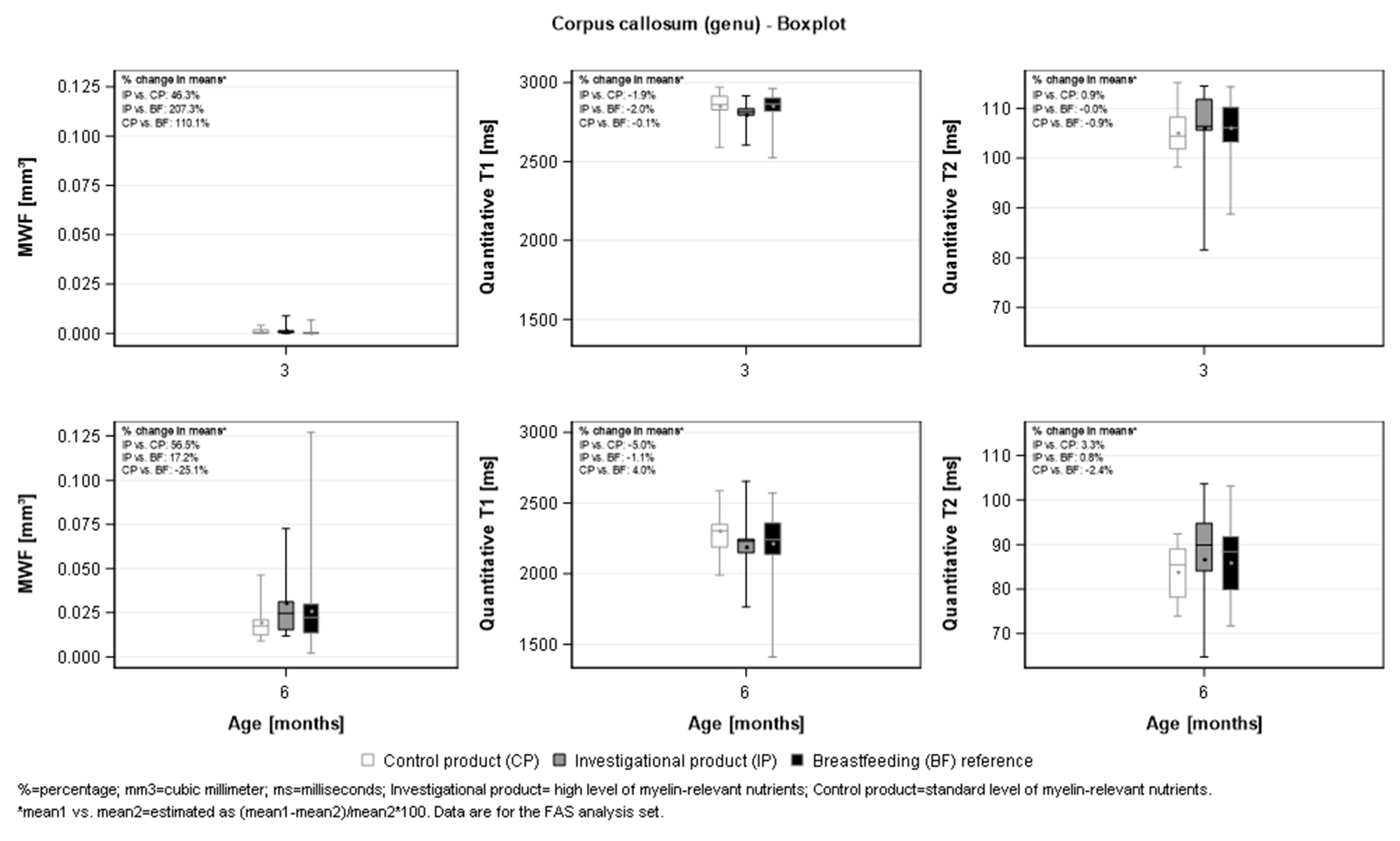

Supplement: Supplementary Figure S6 — Descriptive statistics for myelin volume and structure in the corpus callosum (CC) genu. [file Image_6.JPEG]

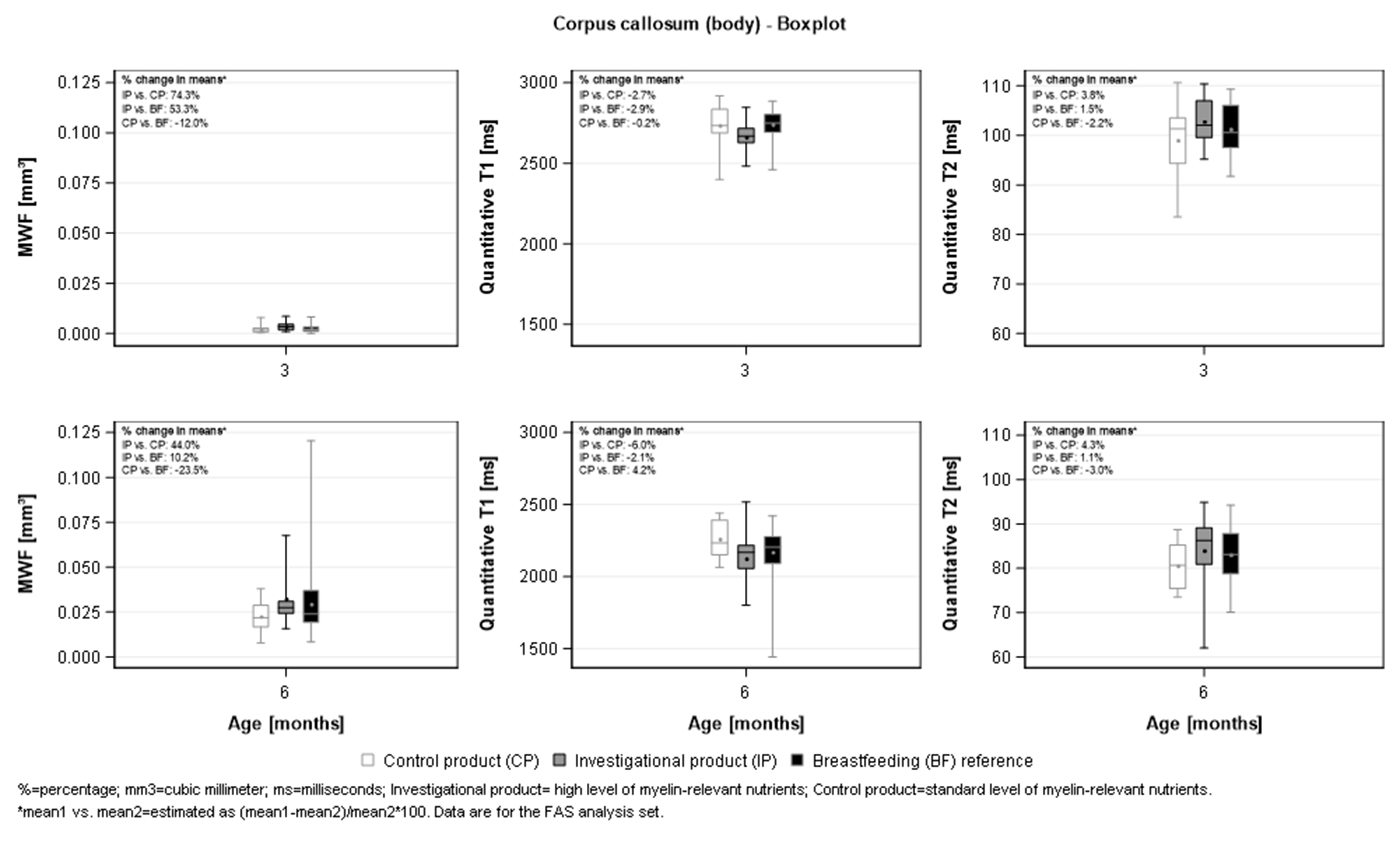

Supplement: Supplementary Figure S7 — Descriptive statistics for myelin volume and structure in the corpus callosum (CC) body. [file Image_7.JPEG]
